# Ultrafast probes of electron–hole transitions between two atomic layers

**DOI:** 10.1038/s41467-018-04291-9

**Published:** 2018-05-10

**Authors:** Xiewen Wen, Hailong Chen, Tianmin Wu, Zhihao Yu, Qirong Yang, Jingwen Deng, Zhengtang Liu, Xin Guo, Jianxin Guan, Xiang Zhang, Yongji Gong, Jiangtan Yuan, Zhuhua Zhang, Chongyue Yi, Xuefeng Guo, Pulickel M. Ajayan, Wei Zhuang, Zhirong Liu, Jun Lou, Junrong Zheng

**Affiliations:** 1 College of Chemistry and Molecular Engineering, Beijing National Laboratory for Molecular Sciences, Peking University, Beijing, 100871 China; 2 Department of Materials Science and NanoEngineering, Rice University, 6100 Main Street, Houston, TX 77005-1892 USA; 3 Beijing National Laboratory for Condensed Matter Physics, CAS Key Laboratory of Soft Matter Physics, Institute of Physics, Chinese Academy of Sciences, Beijing, 100190 China; 4 Department of Chemical Physics, University of Science and Technology of China, Hefei, Anhui 230026 China; 5 Department of Chemistry, Rice University, 6100 Main Street, Houston, TX 77005-1892 USA; 6 State Key Laboratory of Structural Chemistry, Fujian Institute of Research on the Structure of Matter, Chinese Academy of Sciences, Fuzhou, Fujian 350002 China

## Abstract

Phase transitions of electron–hole pairs on semiconductor/conductor interfaces determine fundamental properties of optoelectronics. To investigate interfacial dynamical transitions of charged quasiparticles, however, remains a grand challenge. By employing ultrafast mid-infrared microspectroscopic probes to detect excitonic internal quantum transitions and two-dimensional atomic device fabrications, we are able to directly monitor the interplay between free carriers and insulating interlayer excitons between two atomic layers. Our observations reveal unexpected ultrafast formation of tightly bound interlayer excitons between conducting graphene and semiconducting MoSe_2_. The result suggests carriers in the doped graphene are no longer massless, and an effective mass as small as one percent of free electron mass is sufficient to confine carriers within a 2D hetero space with energy 10 times larger than the room-temperature thermal energy. The interlayer excitons arise within 1 ps. Their formation effectively blocks charge recombination and improves charge separation efficiency for more than one order of magnitude.

## Abstract (web summary)

The investigation into the dynamical transitions of charged quasiparticles on interfaces remains technically challenging. Here, the authors use ultrafast, mid-infrared micro-spectroscopy to unveil the formation of tightly bound interlayer excitons between conducting graphene and semiconducting MoSe_2_.

## Introduction

In optoelectronic and optoelectro-chemical devices composed of semiconductors and conductors, photo excitation can generate transient conducting free carriers by promoting electrons from the occupied valence band into the empty conduction band of the semiconductors^1^. The electrostatic interaction between the depleted valence band states (holes) and excited electrons can lead to the formation of long-lived insulating excitons^2–4^. The evolution of these electron/hole states on interfaces in the devices, which arises from interactions among phonons, photons, and charged quasiparticles, determine their fundamental properties, e.g., luminescence, heat generation, charge separation and transport, redox reaction kinetics, and outcomes^5–7^. In the past, the interfacial dynamics of excited optoelectronic materials were often studied on bulk or nano-sized samples that contain both interfacial and bulk components with absorption, photoluminescence, and visible pump/probe experiments close to the bandgap^1,8,9^. Despite providing a wealth of important information, these techniques are sensitive to creation and destruction of excitons and thus only probe the quasiparticles with negligible center-of-mass momentum because of the small photon momentum. However, the majority of excitons can assume finite momenta as a result of scattering and is optically dark in these measurements^10,11^.

**Fig. 1:**
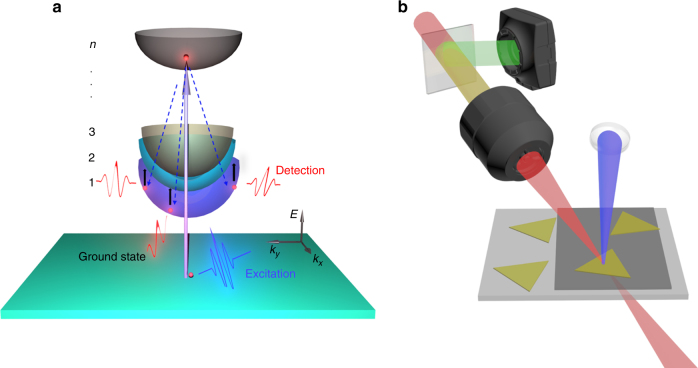
Illustration of experimental setup and principle. **a** Mid-IR probes the excitonic internal quantum transition 1s-2p with a broad momentum distribution. Optical excitation generates free carriers with negligible momentum change. Scattering of free carriers (dash lines) into the exciton states involves large momentum transfers. **b** Illustration of the experimental setup. The IR probe is focused by an objective lens and transmits through the sample, detected by a MCT array detector behind the sample. The reflected visible light through the objective lens is collected by CCD to position the light/matter interaction spot. By tuning the sample stage, heterostructure, graphene, and MoSe_2_ can be respectively studied on the same CaF_2_ substrate. Triangles and the light-black layer represent single-layer MoSe_2_ and graphene, respectively

In contrast, through detecting internal quantum transitions^11–14^, photons with lower energy, e.g., the near- and mid-infrared (IR) or THz, can probe photo-generated excitons at center-of-mass momenta well outside the optically accessible range (Fig. 1a), allowing precise measurements and direct monitoring of excitonic energy and dynamics without interference from other species. The method can effectively avoid mixing together the signals of free carriers, excitons, and defect-trapped carriers that usually occur in visible pump/probe experiments due to the high detection photon energy^15^. On the other hand, the advent of two-dimensional (2D) heterostructures composed of only two atomic layers^16,17^ provides an ideal laboratory for the studies of pure interfacial dynamical transitions of charged quasiparticles. However, limited by spatial resolution, it remains a challenge for regular IR setups to probe electron/hole pairs in 2D heterostructures of which the sizes are typically smaller than the IR beam size. In this work, combining microscope, ultrafast visible/near-infrared/mid-infrared frequency-mixed spectroscopy (Fig. 1b) and state-of-the-art 2D atomic device fabrications, we are able to conduct the first experiments directly monitoring the phase transitions of electron/hole gas between two atomic layers. Studies under various conditions reveal unexpected ultrafast transformation of conducting free carriers into tightly bound insulating interlayer excitons across the conducting graphene and semiconducting MoSe_2_ interface.

## Results

### Photoluminescence of MoSe_2_ is quenched by graphene

**Fig. 2:**
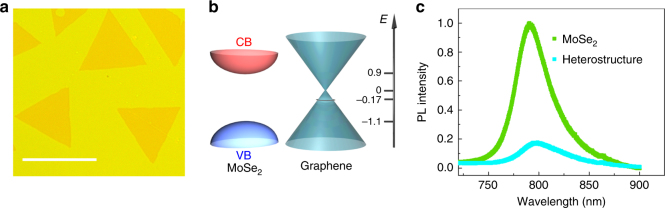
MoSe2/graphene heterostructures. **a** Optical image of the MoSe_2_/graphene heterostructures on a CaF_2_ window. The triangles are MoSe_2_ below a continuous monolayer of graphene. Scale bar represents 100 µm. **b** Band alignment of the heterostructure. The energy levels CBM (conduction band minimum of MoSe_2_), Dirac point and Fermi level of graphene, and VBM (valence band maximum of MoSe_2_) in eV are listed in the order from top to bottom on the right. **c** Photoluminescence (PL) spectra of the MoSe_2_ monolayer and MoSe_2_/graphene heterostructure. PL is severely quenched in the heterostructure

In our experiments, photo-excited electrons/holes are quantum confined within the 2D space of two atomic layers. We investigate heterostructures composed of one monolayer of MoSe_2_ and another monolayer of p-doped graphene (Fig. 2a). The monolayer MoSe_2_ is a direct bandgap semiconductor^2,18^. It has a very small excitonic Bohr radius and anomalously strong interband optical absorptions^2^. Its excitonic binding energy is ~0.6 eV (Supplementary Fig. 24), orders of magnitude larger than bulk materials^12,19,20^. The large binding energy allows the intralayer excitons of MoSe_2_ to remain stable at room temperature. The monolayer graphene is a semi-metallic conductor^21^ in which optical excitations generate free carriers that are rapidly thermalized to reach a new Fermi–Dirac electronic distribution^22^. Because of its gapless band structure, the conducting free carriers in graphene do not transform into insulating excitons^23–25^. The distinctly different opto-electro properties of graphene and MoSe_2_ and other direct bandgap 2D transition metal dichalcogenide (TMDC) materials^2,18^ promise ideal combinations for atomically thin optoelectronic devices in which 2D TMDC functions as photon absorbers and graphene layers serve as transparent electrodes^16,17^.

The band alignment of MoSe_2_/graphene heterostructure is illustrated in Fig. 2b. The separation of conduction band minimum (CBM) and valence band maximum (VBM) of MoSe_2_ is around 2.0 eV^19,20,26^. The Dirac point of graphene is 0.9 eV below CBM of MoSe_2_. The Fermi level of graphene in heterostructure is -0.17 eV, 0.02 eV higher than the monolayer graphene because of charge transfer from the n-doped MoSe_2_. The photoluminescence (Fig. 2c) of the heterostructure at 795 nm (1.56 eV) is dominated by the excitonic 1s transition of MoSe_2_. It is severely quenched by the presence of graphene because of efficient interlayer charge and energy transfers^27^.

### Excitation below MoSe_2_ transition energy

**Fig. 3:**
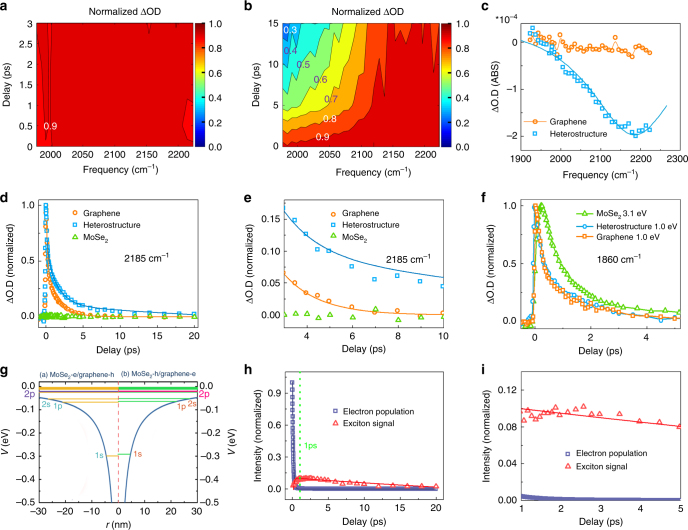
Ultrafast measurements reveal the formation of interlayer excitons. Waiting time-dependent spectra of **a** graphene monolayer and **b** MoSe_2_/graphene heterostructure excited by 1.03 eV photons. The maximum intensity at each waiting time is set to be 1. Thus, the figures only reflect the changes of detection frequency dependence rather than decay dynamics. Each contour represents 10% intensity change. **c** Spectra of MoSe_2_/graphene heterostructure and graphene monolayer at 16 ps after excitation with 1.03 eV photons. The graphene signal (both dots and curve) is already zero, but the heterostructure signal is at peak. The peak is fit with a Lorentzian centered at 2185 cm^−1^ with a width 280 cm^−1^. Dots are data and the curve is calculation. **d** Waiting time-dependent transient IR signals detected at 2185 cm^−1^ of monolayer graphene, MoSe_2_/graphene heterostructure, and MoSe_2_ monolayer with 1.03 eV excitation. The dynamics of the heterostructure is apparently slower than graphene. Dots are experimental data, and lines are theoretical calculations. The negligibly small signal of MoSe_2_ is normalized to the maximum intensity of the graphene signal. **e** Enlarged waiting time-dependent transient IR signals after 3 ps in Fig. 3d, which illustrates the nonzero tail of the heterostructure signal. **f** Waiting time-dependent transient IR signals detected at 1860 cm^−1^ of the monolayer graphene and MoSe_2_/graphene heterostructure with 1.03 eV excitation, and MoSe_2_ monolayer with 3.1 eV excitation. Different from the 2185 cm^−1^ detection, within experimental uncertainty the dynamics of graphene and heterostructure are the same. Both are slower than the free-carrier dynamics in MoSe_2_ monolayer with 3.1 eV excitation. **g** Calculated interlayer exciton energy levels of MoSe_2_/graphene heterostructure with graphene’s Fermi level at -0.17 eV with a 2D model. The calculations show a binding energy of about 0.3 eV for the interlayer excitons. **h** Calculated waiting time dependence of electronic dynamics (navy) for graphene with Fermi level at −0.17 eV, and interlayer exciton signal (red). Lines are kinetic analyses. **i** Calculated huge population difference between excitons and free carrier after 1 ps in Fig. 3h, when the concentration of free carriers is close to 0

In ultrafast experiments (Fig. 1b), the sample is excited with 40-femtosecond near infrared and blue pulses at 1200 nm (1.03 eV, below the MoSe_2_ bandgap) and 400 nm (3.1 eV), respectively. The optical density change (proportional to the optical conductivity change, Supplementary Equation 5) following excitation is monitored with a mid-IR pulse. Figure 3a, b display optical density changes of a monolayer graphene and a graphene/MoSe_2_ heterostructure detected in the mid IR range 1950–2230 cm^−1^ following excitation at 1.03 eV. Transient spectra and kinetic data are provided in SI (Supplementary Fig. 1). To illustrate the changes of detection frequency dependence in the samples, the maximum intensity at each waiting time is set to be 1. The spectrum of monolayer graphene remains flat across the entire detection frequency range and independent of waiting time (Fig. 3a). In contrast, the spectrum of the heterostructure is time dependent. With the increase of delay time, the relative intensity in low frequencies drops significantly (Fig. 3b). The spectra at 16 ps (Fig. 3c) reveal the striking difference between heterostructure and graphene: a resonant peak centered at 2185 cm^−1^ (0.27 eV) with a Lorentzian width of 280 cm^−1^ remains in heterostructure that lasts for more than 20 ps (Fig. 3c) while the graphene signal is already zero. The huge signal difference can be further revealed by the detection frequency dependent dynamics. Detected at 2185 cm^−1^ (Fig. 3d), the heterostructure dynamics is apparently slower than graphene and with a nonzero tail (Fig. 3e). However, at a lower detection frequency, 1860 cm^−1^ (Fig. 3f), the nonzero tail of the heterostructure signal disappears, and its dynamics becomes faster. In fact, at this detection frequency, the heterostructure and graphene follow essentially the same dynamics, and both are slower than the intralayer free-carrier dynamics^4^ in monolayer MoSe_2_ excited with 3.1 eV photons. No signal is observed for MoSe_2_ monolayer with the 1.03 eV excitation. The signal of monolayer MoSe_2_ excited with 3.1 eV photons rises apparently slower than those of samples excited with 1.03 eV photons. The difference mainly originates from different signal mechanisms. The signal with the 3.1 eV excitation directly comes from the absorptions of both fast generated free carriers and excitons generated in a subsequent slower process, but the signal with 1.03 eV excitation is from the very fast electronic thermal redistribution in graphene as discussed in the following.

### Interlayer excitonic internal 1s-2p quantum transition

The huge difference in signal reveals distinct evolutions of electrons/holes in graphene and heterostructure. The excitation photon energy 1.03 eV is lower than the excitonic transition energy (1.59 eV) of MoSe_2_, and thus negligible electron/hole pairs are generated in the monolayer MoSe_2_. In the monolayer graphene, the excitation generates $5.2 \times 10^{12}$electrons-holes/cm^2^ with energy separation equally above and below the Dirac point. Energy rapidly thermalizes among the charge carriers and relaxes through electron/phonon couplings with the transition matrix element^28,29^

1 $$M_{{\mathbf{k}}\prime,{\mathbf{k}}}^{({\mathrm{TO}}\& {\mathrm{LO}})} \approx 3\eta \sqrt {\frac{\hbar }{{4M_C\omega _{{\mathrm{phonon}}}}}}$$

where *η* is the electron–phonon coupling parameter, $\omega _{{\mathrm{phonon}}}$ is the phonon angular frequency, and *M*_C_ is the mass of a carbon atom. The processes cause an electronic temperature change and a Fermi–Dirac electronic population redistribution, leading to the optical conductivity change that is determined by both intraband and interband transitions^30^:

2 $$\begin{array}{l}\sigma _{{\mathop{\rm{int}}} {\mathrm{er}}}\left( \omega \right) = i\frac{{e^2\hbar \omega }}{{\pi \hbar }}{\int}_0^{ + \infty } {{\mathrm{d}}\varepsilon \frac{1}{{(2\varepsilon )^2 - (\hbar \omega + i\Gamma )^2}}\left[ {f_{{\mathrm{FD}}}(\varepsilon - \mu ) - f_{{\mathrm{FD}}}( - \varepsilon - \mu )} \right]} \\ \sigma _{{\mathrm{intra}}}\left( \omega \right) = i\frac{{e^2/\pi \hbar }}{{\hbar \omega + i\hbar /\tau _e}}{\int}_0^{ + \infty } {{\mathrm{d}}\varepsilon \left[ {f_{{\mathrm{FD}}}(\varepsilon - \mu ) + 1 - f_{{\mathrm{FD}}}( - \varepsilon - \mu )} \right]} \end{array}$$

where *f*_FD_ is the Fermi–Dirac distribution function, *μ* is the chemical potential (Fermi energy), and *e* is the elementary charge, *Γ* is the broadening of the interband transitions, whereas *τ*_*e*_ is the relaxation time due to intraband carrier scattering. Quantitative analyses of the graphene signal according to Eqs. 1 and 2 describe the experimental results very well (Fig. 3d), yielding a Fermi level of −0.19 eV, the electron/hole population dynamics, and the temperature dynamics of graphene. Details of the analyses are provided in SI. The decay of free electrons/holes in graphene is extremely fast, with a time constant of 120 fs (Fig. 3h, i, navy). These obtained graphene properties are similar to the previously reported results^22,31^.

In the heterostructure, the detection frequency dependent signals and dynamics (Fig. 3b–f) resemble those free carrier/exciton transitions of GaAs quantum wells observed in the THz range, where the resonant peak is assigned to the excitonic internal quantum 1s-2p transition^12,32^. A similar 1s-2p transition peak was also observed in the mid-IR range for monolayer WSe_2_ on a diamond^11^. In this work, different from the two previous experiments, the graphene/MoSe_2_ heterostructure sample is not a pure substance, but a combination of two monolayers. Thus, the nature of the exciton that give rise to the resonant peak at 2185 cm^−1^ can have several possible origins: 1, the internal neutral and charged excitons, e.g., regular excitons and trions^33^ in graphene; 2, the internal neutral and charged excitons in MoSe_2_; and 3, the interlayer neutral and charged excitons.

In the following, combining experimental data and literature, neutral interlayer excitons are concluded to be the dominant species that produce the resonant signals. First of all, the contribution of the charged excitons (including trions and other excitons composed of more than three carriers) is very minor in room temperature optical/IR pump/probe experiments. This has been completely addressed in the SI of the previous work^11^. In brief, the binding energy of trions is only about 30 meV^33^. The binding energy of excitons with more particles is even smaller. At room temperature, where our experiments were carried out, most of the trions had dissociated due to thermal motions^11,34^, which has been verified with temperature-dependent photoluminescence^11,34^. Second, the intralayer exciton in graphene is not likely. Although the carriers in doped graphene are no longer massless due to the Fermi level shift from the Dirac point and a slight symmetry breaking^35^, tightly bound excitons cannot form because the bandgap of graphene in the heterostructure is too small (4.4 meV, Supplementary Fig. 25), compared to room temperature thermal energy (26 meV)^35^. Third, intralayer excitons in MoSe_2_ (with interlayer effects that cause its binding energy to drop) are not likely either. The combination of the experiments with 3.1 and 1.03 eV excitations in Fig. 3d, f rule out this possibility. 1.03 eV is significantly lower than the bandgap of MoSe_2_. Excitation with it cannot produce detectible amounts of free carriers or excitons in MoSe_2_ (green dots in Fig. 3d). In the heterostructure, if the peak at 2185 cm^−1^ (Fig. 3c) was due to the intralayer excitons in MoSe_2_, the excitons would come from the holes and electrons transferred from graphene. After the carriers transfer to MoSe_2_, they would behave like free carriers^4^, and then these free carriers would decay to form excitons and some possible trapped states inside MoSe_2_. In the process, the free-carrier dynamics inside MoSe_2_ would be similar to, or slightly slower (due to lower energy)^4^ than the free carriers created with 3.1 eV excitation in the monolayer MoSe_2_. However, experimentally, the free-carrier dynamics detected at 1860 cm^−1^ of the heterostructure excited with 1.03 eV is significantly faster than that inside the monolayer MoSe_2_, and essentially the same as graphene (Fig. 3f). The detection energy 1860 cm^−1^ is lower than the excitonic internal quantum transition energy, 2185 cm^−1^. Therefore, signals at 1860 cm^−1^ cannot probe excitons, but only reflect free-carrier dynamics. This comparison of free-carrier dynamics rules out the possibility that the observed dynamics of the heterostructure is mainly from MoSe_2_ intralayer dynamics, indicating that the resonant peak at 2185 cm^−1^ should not belong to MoSe_2_ intralayer excitons. In fact, the intralayer excitonic internal quantum transition of monolayer MoSe_2_ appears at a much higher energy, 0.55 eV (Supplementary Fig. 24), further supporting that the resonant peak at 2185 cm^−1^ observed in the heterostructure should not belong to the MoSe_2_ intralayer excitons. In conclusion, all these systematic experimental results suggest that the neutral interlayer excitons are the dominant species leading to the resonant peak at 2185 cm^−1^. The similarity of the free-carrier dynamics between heterostructure and graphene (Fig. 3f) is interesting and worth further discussion. A similar phenomenon in a WS_2_/MoS_2_ heterostructure was also observed in our previous work^4^ in the formation process of interlayer excitons, the free-carrier dynamics inside the heterostructure follow the faster dynamics (MoS_2_) of the two monolayers. It is conceivable that the formation of interlayer excitons need carriers to diffuse in both the layers and the faster partner dominates.

However, the interlayer excitons involving graphene with binding energy larger than 2185 cm^−1^ (0.27 eV) seem to be unlikely and completely unexpected if estimated with conventional hydrogen-like 3D model. It is well known that pristine graphene has massless carriers, which cannot form excitons^36^. In most CVD grown graphene, which is effectively doped, like the one used in our experiments, the Fermi level is not at the Dirac point. Because of the Fermi level shift and the slight symmetry breaking due to the formation of heterostructure^35^, the carriers are no longer massless (Supplementary Fig. 25). The effective mass of the carriers in graphene with Fermi level −0.17 eV is between 0.01–0.03 *m*_0_ (*m*_0_, the free electron mass), similar to 0.012 *m*_0_ reported for an epitaxy-grown graphene^37^. Estimated with this effective mass and the conventional 3D hydrogen-like model in which the binding energy is linearly proportional to the reduced mass, the binding energy of graphene/MoSe_2_ would be only 1/60 of the MoSe_2_ intralayer excitons. The binding energy of MoSe_2_ intralayer excitons has been computed to be between 0.4 and 0.6 eV^19^. Our experiments (Supplementary Fig. 24) also verify that it is ~0.60 eV, under our experimental conditions. This would suggest that the estimated binding energy of the interlayer excitons should be smaller than 0.01 eV, significantly smaller than 0.27 eV, the observed excitonic internal quantum transition energy. The key to solve the contradiction lies in the fact that the atomic layer 2D excitons cannot be described by 3D hydrogen-like models^38,39^. Instead, the 2D models must be applied^38,39^. In the 2D models, the binding energy is no longer linearly proportional to the effective mass^38,39^. Estimated from a 2D model, the interlayer excitonic binding energy is much larger, about 1/2–1/4 of the MoSe_2_ intralayer excitonic binding energy, which is around 0.1–0.3 eV. This estimated value is close to the excitonic internal quantum transition energy 0.27 eV observed. Using a 2D model that has been previously tested on other 2D materials^4,38^, the binding energy of MoSe_2_/graphene interlayer excitons is calculated to be 0.3 eV (Fig. 3g). The calculations match the experimental results very well. Calculation details are in SI (Supplementary Figs 8–23).

In summary, the excitation of MoSe_2_/graphene heterostructure with 1.03 eV photons results in the formation of interlayer excitons. The dynamical process can be visualized in terms of free-carrier/exciton transitions. The photon excitation creates free carriers in graphene. The semi-instantaneous thermalization of carriers^22^ leads to a new Fermi–Dirac distribution, in which the number of holes at energy below the VBM of MoSe_2_ is around four times more than that of electrons above the CBM of MoSe_2_. These charged carriers rapidly transfer between the two layers^4,27^ and reach quasi-equilibrium, finally resulting in holes dominantly in MoSe_2_ and electrons in graphene. Oppositely charged quasiparticles on the two layers attract each other and form interlayer excitons. The excitonic 1s-2p transition leads to the appearance of a resonant peak centered at 2185 cm^−1^ (0.27 eV) (Fig. 3c). As shown in Fig. 3h (navy line vs red curve), the interlayer exciton’s lifetime is much longer than the free carriers, similar to that of WS_2_/MoS_2_ heterostructure^4^. At time zero, most electrons/holes in the heterostructure are free carriers, while within 1 ps the majority of the remaining electron/hole pairs are already interlayer excitons (Fig. 3i). Kinetic analyses of the experimental results normalized with optical cross sections show that the interlayer exciton has a formation time constant 0.49 ± 0.27 ps, with a lifetime 65 ± 20 ps (red in Fig. 3h, dots-experiments and line-theory). The results indicate that around $25\%$ (120 fs/490 fs) of the free carriers’ photogenerated in graphene transform into interlayer excitons within hundreds of fs, and by the time the majority of other free carriers have recombined and released heat in graphene. Calculation details and more discussions are provided in SI (Supplementary Figs 1–7). The surprising charge flow suggested by ultrafast measurements is consistent with photocurrent measurements, in which the photocurrent is measured between graphene on the top of MoSe_2_ and graphene on the top of Si. As displayed in Supplementary Fig. 27 (with a thin layer of SiO_2_), the 980 nm photoexcitation, which excites only graphene but not MoSe_2_, produces external photocurrent flowing from the portion of graphene that is on the top of Si (with a thin layer of SiO_2_) to that on the top of MoSe_2_, which is a clear signal that the quasi-equilibrium of MoSe_2_ (hole)/graphene (electron) inside the heterostructure is reached by the 980 nm photoexcitation. The results have a very interesting indication: under the experimental condition, graphene functions as a photon absorber and MoSe_2_ serves as a charge acceptor, rather than their traditional roles, in which graphene is the transparent electrode and MoSe_2_ is the chromophore.

### Excitation above MoSe_2_ transition energy

**Fig. 4:**
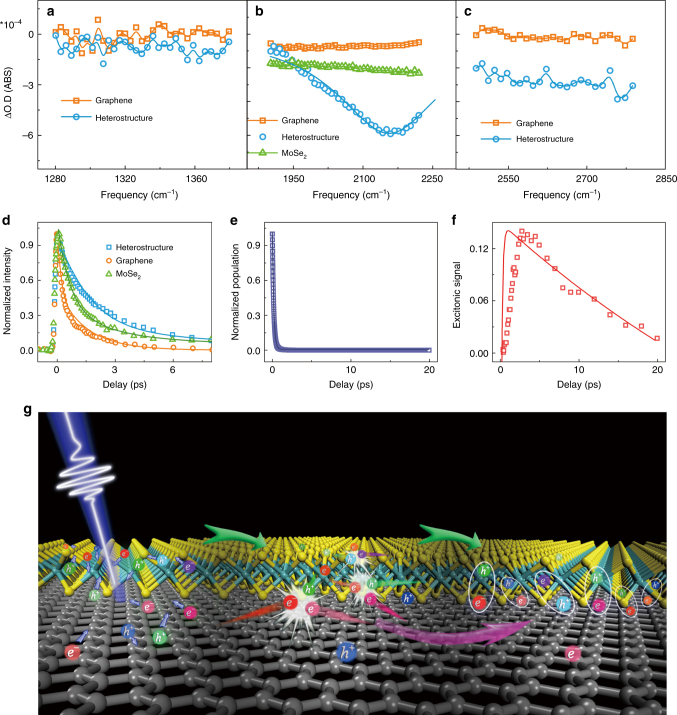
Excitation with 3.1 eV also leads to the formation of interlayer excitons. **a** Spectra of MoSe_2_/graphene heterostructure and graphene monolayer at 16 ps after excitation with 3.1 eV photons detected in the frequency range 1280–1380 cm^−1^ below the excitonic 1s-2p transition frequency. Both signals are zero. **b** Spectra of MoSe2/graphene heterostructure, MoSe_2_, and graphene at 16 ps after excitation with 3.1 eV photons detected in the frequency range 1900–2230 cm^−1^ covering the excitonic 1s-2p transition frequency. Both graphene and MoSe_2_ spectra are flat, whereas that of heterostructure is a peak with a much higher intensity centered at 2156 cm^−1^, with a Lorentzian width 278 cm^−1^. **c** Spectra of MoSe_2_/graphene heterostructure and graphene monolayer at 16 ps after excitation with 3.1 eV photons detected in the frequency range 2450–2800 cm^−1^, above the excitonic 1s-2p transition frequency. The graphene signal is zero, and that of the heterostructure is a nonzero line because of the transition to higher bound and unbound states12. **d** Waiting time-dependent normalized transient IR signals detected at 2185 cm^−1^ of MoSe_2_/graphene heterostructure, MoSe_2_, and graphene. The dynamics of heterostructure is the slowest. The initial absolute intensity ratio of the three samples is 3.4/1.8/1=heterostructure/graphene/MoSe_2_. Dots are data, and lines are theoretical calculations. **e** The electronic dynamics in graphene of heterostructure. Dots are calculations and the line is fitting. **f** The interlayer excitonic signal in the heterostructure. Dots are experimental data and the line is kinetic calculation. **g** Illustration of electron/hole gas transition in the heterostructure. An electron/hole pair in ellipse represents an exciton. Excitation with photo energy (3.1 eV) higher than MoSe2 bandgap creates free carriers in both MoSe_2_ and graphene. The carriers transfer between the two layers. The carriers collide with each other and transfer energy and momenta so that phonon motions are not necessary for the ultrafast formation of interlayer excitons. Because of the band alignment, more electrons are on the graphene side and more holes are on the MoSe_2_ side

The ultrafast formation of interlayer excitons is also observed when the majority of free carriers is from MoSe_2_. Figure 4a–c displays the spectra of graphene, MoSe_2_ and MoSe_2_/graphene heterostructure detected in three different IR ranges at 16 ps after excitation with 3.1 eV photons. In the low-frequency range (1280–1380 cm^−1^), signals of both graphene and heterostructure are zero. Between 1900 and 2230 cm^−1^, the spectrum of graphene is very close to zero, whereas the spectrum of the heterostructure is at peak. In the high frequency range (2500–2800 cm^−1^), the signal of graphene remains zero, but that of heterostructure is a flat spectrum with an amplitude of about 30–40% of the peak value at 2156 cm^−1^. Similar to those following 1.03 eV excitation (Fig. 3c), the peak centered at 2156 cm^−1^ with a width 278 cm^−1^ is attributed to the interlayer excitonic transition^12,32^. The central frequency slightly redshifts from 2185 cm^−1^ with 1.03 eV excitation. This frequency difference is probably because of the experimental uncertainty, rather than excitonic difference. Our frequency resolution is about 9 cm^−1^, and the broadband super continuum probe pulse has a spatial dependence of frequency distribution that can lead to the signal intensity at a certain frequency, dependent on the focus condition. These two factors together can cause the peak frequency to shift for 10% of its width. Therefore, within the experimental uncertainty, we would conclude that the peak frequency and width are the same as those excited with 1.03 eV photons. The flat spectrum at higher frequencies corresponds to transitions into higher-energy bound and continuum states^12,32^. The insulating nature of the interlayer exciton is apparent from the vanishing optical conductivity at low frequencies (Fig. 4a).

3.1 eV is higher than the bandgap of MoSe_2_. In the heterostructure, the photo excitations not only generate free carriers in graphene, but also promote electrons into the unbound continuum of MoSe_2_. The excited quasiparticles exchange between the two monolayers, resulting in electrons dominantly in graphene and holes mainly in MoSe_2_ which attract each other across layers. Such a charge separation produces a photocurrent, in which the electrons flow from graphene to MoSe_2_ in the external circuit. The photocurrent results are shown in Supplementary Fig. 28.

Because the absorption of MoSe_2_ (14%) is significantly higher than that of graphene (4.14%) at 3.1 eV^40–42^, overall the interlayer charge transfers result in higher electronic temperature in graphene. Excluding the free carriers remaining in MoSe_2_, the effect is around equal to 200% excitation flux increase for graphene monolayer, assuming semi-instantaneous interlayer charge transfers^4,27^ and electronic thermalization^22^. Our calculations show that the temperature increase with additional 200% flux slows the graphene electronic dynamics for about 30%, from 130 to 170 fs (Supplementary Fig. 5). The temperature effect together with the formation of long-lived interlayer excitons causes the dynamics of the heterostructure to be significantly slower than the monolayer graphene (Fig. 4d). The heterostructure dynamics is even slower than that of MoSe_2_ monolayer, which only reflects the dynamics of the free carriers in MoSe_2_^4^. Kinetic analyses show that the interlayer exciton formation time constant is 0.51 ± 0.28 ps, with an excitonic lifetime 55 ± 20 ps (red in Fig. 4e, dots-experiments and line-theory.), indicating around $33\%$ of the free carriers photogenerated form the interlayer excitons. The exciton dynamics are similar to excitation with 1.03 eV photons. However, because the free-carrier dynamics is slower with 3.1 eV excitation, more excitons are generated.

## Discussion

The fast formation of MoSe_2_/graphene interlayer excitons within hundreds of fs is surprising. On one hand, previous theories of exciton formation consider only the extreme dilute limit, where independent charge carriers interact with phonons^43,44^, predicting much slower ps to hundreds of ps exciton formation dynamics. On the other hand, the existence of interlayer excitons is not expected in a semiconductor/graphene heterostructure, where the Fermi level of graphene typically lies between VBM and CBM of the semiconductor layer^45^, because the aforementioned carriers in graphene is nearly massless, and the ultrafast (<50 fs) interlayer charge transfers^4,27^ send both electrons and holes into graphene^45^, where they recombine within a couple of hundred fs^23,25^. The commonly accepted but oversimplified pictures, however, cannot account for the complex many-body dynamics in real systems and our experiments. Quasiparticles that excitons are composed of cannot be treated separately. In a photo-excited electron/hole gas, the ultrafast Coulomb interactions result in fundamentally new properties through pairing and higher-order correlations^46^. Some of these can be intuitively visualized by processes such as a pair of free electron and hole forming an exciton by transferring their energy and momenta to other free carriers or redistributions among pairs through collisions (Fig. 4g). The many-body Coulomb interactions naturally explain the ultrafast formation of interlayer excitons observed in our experiments. Once electrons and holes form excitons, they cannot recombine directly because of the in-plane momentum mismatch that is due to the random relative orientation between graphene and MoSe_2_. The carriers need to either compensate for the momentum mismatch or cross the binding barrier to recombine, which takes extra steps and time. Therefore, the formation of interlayer excitons effectively retains the charge separation across the interface of the heterostructure by significantly slowing down the otherwise ultrafast interlayer charge transfers.

The transition of conducting free carriers into insulating interlayer excitons observed in the semiconductor/semimetallic 2D heterostructure reveals the significance of many-body interactions in mediating and thus producing sophisticated electronic dynamics and states on interfaces. The unexpected formation of the tightly bound interlayer excitons suggest that carriers in the doped graphene of the heterostructure is no longer massless, and an effective mass as small as one or two percent of *m*_0_ is sufficient to confine carriers within a 2D hetero space with energy 10 times larger than the thermal energy at room temperature. One important consequence of the ultrafast formation of interlayer excitons is that it significantly improves the intrinsic charge separation efficiency on graphene interface. Without interlayer excitons, the photo-generated carriers would transfer to and recombine in graphene within a couple of hundred fs. The long-lived interlayer excitons (>50 ps) keep the charged quasiparticles on different layers for more than 20 times longer. The greatly improved intrinsic charge separation efficiency is important for many applications that utilize the conversion of photo energy into electronic energy or electricity, e.g., solar cells, photo-detections, and photo-electrochemistry. We anticipate that the microspectroscopic IR response uncovered here will enable realtime and precise studies of the new non-equilibrium states on atomic interfaces that are critical for future developments and applications of atomically thin and other optoelectronic devices, but cannot be studied with other means.

## Methods

### MoSe_2_ monolayer growth

We follow the CVD method of growing high-quality monolayer MoSe_2_ in our prior publication^47^ using Selenium pellets and Molybdenum oxide as sources.

### Graphene monolayer growth

CVD method was used to grow graphene on an electropolished copper foil with methane as a precursor. The copper foil was annealed at 1000 ℃ for 20 min, followed by graphene growth for 9 min using 3.5 sccm of methane under the same temperature. 15% H_2_/Ar was used as a carrier gas and the pressure was kept around 1 torr during the whole process.

### Heterostructure preparation

The as-grown MoSe_2_ are transferred onto the CaF_2_ substrates for transmission mode laser experiments. The process is: at first, a thin layer of PMMA (PMMA-B4 4%wt) is spin-coated onto the sample on SiO_2_ substrate twice at the speed of 4000 rpm, and then the sample is carefully placed in a Buffer HF (1:5) and allowed to float. After 20 h, the PMMA sheets are separated from the SiO_2_ substrate and fished with glass plate, and then moved to Di-water for rinsing for a few times. The pre-cleaned CaF_2_ windows are used to fish the PMMA sheets. The CaF_2_ windows with PMMA and samples stay in room temperature and vacuum for 24 h. Finally, acetone is used to remove the PMMA. The MoSe_2_ triangles on the CaF_2_ substrate are confirmed by optical microscope. Graphene on the Cu foils spun with PMMA is dissolved in Cu_2_Fe_3_, and the PMMA sheet is fished and transferred like the same method above onto the MoSe_2_ monolayers. PMMA is removed with acetone. Finally, the sample is annealed in vacuum at 300 °C for 3 h.

### Raman and photoluminescence measurements

Raman and PL spectroscopy were carried out using a Horiba Jobin Yvon LabRAM HR-Evolution Raman microscope. The excitation light is a 532 nm laser, with an estimated laser spot size of 1 μm and the laser power of 1 mW.

### Ultrafast visible-NIR/infrared microspectroscopy

The experimental setup^4^ of the ultrafast visible-NIR/infrared microspectroscopy is illustrated in Fig. 1b. Briefly, the output of a femtosecond amplifier laser system (at a repetition rate of 1 kHz, 1.6 mJ energy per pulse, 800 nm central wavelength, and a pulse duration of ∼40 fs, Uptek Solutions Inc.) is split into two parts. One is used to pump a home-built nonlinear optical parametric amplifier to generate visible and near-IR-1 laser pulses with tunable wavelengths, and the other is directed to generate an ultra-broadband super-continuum pulse, which covers almost the whole mid-IR region^48,49^ or a near-IR-2 pulse. In ultrafast experiments, the visible or NIR-1 pulse is the pump light with the central wavelength and excitation power adjusted based on need. The interaction spot on the samples varies from 120 to 250 micron. The mid-IR super-continuum pulse or NIR-2 pulse acts as the probe light, which is focused at the sample by the reflective objective lens (15X/0.28NA, Edmund Optics Inc.) to reduce the spot size to the level of the sample area (<40 μm). A 300-megapixel microscope digital camera is used to align the pump/probe beam to proper sample area. The probe light is detected by a liquid-nitrogen-cooled mercury-cadmium-telluride (MCT) array detector or InSb detector after frequency resolved by a spectrograph with a resolution of 1–3 cm^−1,^ which is dependent on the central frequency. The time delay between the pump light and probe light is controlled by a motorized delay stage.

### Data availability

The data that support the findings of this study are available from the corresponding author upon request.

## Electronic supplementary material

Supplementary Information

Peer Review File

## References

[1] Chemla DS, Shah J (2001). Many-body and correlation effects in semiconductors. Nature.

[2] Mak, K. F., Lee, C., Hone, J., Shan, J., Heinz, T. F. Atomically thin MoS2: a new direct-gap semiconductor. *Phys. Rev. Lett*. **105**, 136805 (2010). 10.1103/PhysRevLett.105.136805 21230799

[3] Ye ZL (2014). Probing excitonic dark states in single-layer tungsten disulphide. Nature.

[4] Chen HL (2016). Ultrafast formation of interlayer hot excitons in atomically thin MoS2/WS2 heterostructures. Nat. Commun..

[5] Vandewal K (2014). Efficient charge generation by relaxed charge-transfer states at organic interfaces. Nat. Mater..

[6] Schmidt H, Giustiniano F, Eda G (2015). Electronic transport properties of transition metal dichalcogenide field-effect devices: surface and interface effects. Chem. Soc. Rev..

[7] Hagfeldt A, Gratzel M (2000). Molecular photovoltaics. Acc. Chem. Res..

[8] Heeger AJ (2014). 25th Anniversary article: bulk heterojunction solar cells: understanding the mechanism of operation. Adv. Mater..

[9] Kraabel B, McBranch D, Sariciftci NS, Moses D, Heeger AJ (1994). Ultrafast spectroscopic studies of photoinduced electron-transfer from semiconducting polymers to c-60. Phys. Rev. B.

[10] Marie X, Urbaszek B (2015). 2D materials ultrafast exciton dynamics. Nat. Mater..

[11] Poellmann C (2015). Resonant internal quantum transitions and femtosecond radiative decay of excitons in monolayer WSe2. Nat. Mater..

[12] Kaindl RA, Carnahan MA, Hagele D, Lovenich R, Chemla DS (2003). Ultrafast terahertz probes of transient conducting and insulating phases in an electron-hole gas. Nature.

[13] Cha S (2016). 1s-intraexcitonic dynamics in monolayer MoS2 probed by ultrafast mid-infrared spectroscopy. Nat. Commun..

[14] Steinleitner P (2017). Direct observation of ultrafast exciton formation in a monolayer of WSe2. Nano Lett..

[15] Wang HN, Zhang CJ, Rana F (2015). Ultrafast dynamics of defect-assisted electron hole recombination in mono layer MoS2. Nano Lett..

[16] Britnell L (2013). Strong light-matter interactions in heterostructures of atomically thin films. Science.

[17] Geim AK, Grigorieva IV (2013). Van der Waals heterostructures. Nature.

[18] Bhimanapati GR (2015). Recent advances in two-dimensional materials beyond graphene. ACS Nano.

[19] Berkelbach, T. C., Hybertsen, M. S. and Reichman, D. R. Theory of neutral and charged excitons in monolayer transition metal dichalcogenides. *Phys. Rev. B* **88**, 045318 (2013).

[20] Ugeda MM (2014). Giant bandgap renormalization and excitonic effects in a monolayer transition metal dichalcogenide semiconductor. Nat. Mater..

[21] Novoselov KS (2012). A roadmap for graphene. Nature.

[22] Malard, L. M., Mak, K. F., Neto, A. H. C., Peres, N. M. R. & Heinz, T. F. Observation of intra- and inter-band transitions in the transient optical response of graphene. *New. J. Phys*. **15**, 015009 (2013).

[23] Yang, L. Excitons in intrinsic and bilayer graphene. *Phys. Rev. B* **83**, 085405 (2011).

[24] Mak, K. F., Shan, J. & Heinz, T. F. Seeing many-body effects in single- and few-layer graphene: observation of two-dimensional saddle-point excitons. *Phys. Rev. Lett.* **106**, 046401 (2011). 10.1103/PhysRevLett.106.046401 21405342

[25] Fallah, F. & Esmaeilzadeh, M. Energy levels of exciton in a gapped graphene sheet. *J. Appl. Phys.* **114**, 073702 (2013).

[26] Gong. C. et al. Band alignment of two-dimensional transition metal dichalcogenides: application in tunnel field effect transistors. *Appl. Phys. Lett.* **103**, 053513 (2013).

[27] Hong XP (2014). Ultrafast charge transfer in atomically thin MoS2/WS2 heterostructures. Nat. Nanotechnol..

[28] Li, Z. Z., Wang, J. Y. & Liu, Z. R. Intrinsic carrier mobility of Dirac cones: the limitations of deformation potential theory. *J. Chem. Phys.* **141**, 144107 (2014). 10.1063/1.4897533 25318715

[29] Park CH (2014). Electron-phonon interactions and the intrinsic electrical resistivity of graphene. Nano. Lett..

[30] Dawlaty, J. M. et al. Measurement of the optical absorption spectra of epitaxial graphene from terahertz to visible. *Appl. Phys. Lett.* **93**, 196405 (2008).

[31] Zhang, W. J. et al. Ultrahigh-gain photodetectors based on atomically thin graphene-MoS2 heterostructures. *Sci. Rep.* **4**, 3826 (2014). 10.1038/srep03826 PMC3899643 24451916

[32] Kaindl, R. A., Hagele, D., Carnahan, M. A., Chemla, D. S. Transient terahertz spectroscopy of excitons and unbound carriers in quasi-two-dimensional electron-hole gases. *Phys. Rev. B* **79**, 045320 (2009).

[33] Mak KF (2013). Tightly bound trions in monolayer MoS2. Nat. Mater..

[34] Wang G (2014). Valley dynamics probed through charged and neutral exciton emission in monolayer WSe2. Phys. Rev. B.

[35] Ma YD (2011). First-principles study of the graphene@MoSe2 heterobilayers. J. Phys. Chem. C.

[36] Castro Neto AH, Guinea F, Peres NMR, Novoselov KS, Geim AK (2009). The electronic properties of graphene. Rev. Mod. Phys..

[37] Tiras E (2013). Effective mass of electron in monolayer graphene: Electron-phonon interaction. J. Appl. Phys..

[38] Zhu XY (2015). Charge transfer excitons at van der Waals interfaces. J. Am. Chem. Soc..

[39] Olsen T, Latini S, Rasmussen F, Thygesen KS (2016). Simple screened hydrogen model of excitons in two-dimensional materials. Phys. Rev. Lett..

[40] Liu HL (2014). Optical properties of monolayer transition metal dichalcogenides probed by spectroscopic ellipsometry. Appl. Phys. Lett..

[41] Mak KF, Shan J, Heinz TF (2011). Seeing many-body effects in single- and few-layer graphene: observation of two-dimensional saddle-point excitons. Phys. Rev. Lett..

[42] Beal AR, Liang WY, Knights JC (1972). Transmission spectra of some transition-metal dichalcogenides .2. group via - trigonal prismatic coordination. J. Phys. C Solid State Phys..

[43] Selbmann PE, Gulia M, Rossi F, Molinari E, Lugli P (1996). Coupled free-carrier and exciton relaxation in optically excited semiconductors. Phys. Rev. B.

[44] Siantidis, K., Axt, V. M. & Kuhn, T. Dynamics of exciton formation for near band-gap excitations. *Phys. Rev. B* **65**, 035303 (2002).

[45] He JQ (2014). Electron transfer and coupling in graphene-tungsten disulfide van der Waals heterostructures. Nat. Commun..

[46] You Y (2015). Observation of biexcitons in monolayer WSe2. Nat. Phys..

[47] Wang XL (2014). Chemical vapor deposition growth of crystalline mono layer MoSe2. ACS Nano.

[48] Chen H (2013). Molecular conformations of crystalline L-cysteine determined with vibrational cross angle measurements. J. Phys. Chem. B.

[49] Chen H (2013). Vibrational cross-angles in condensed molecules: a structural tool. J. Phys. Chem. A.

