# Author Correction: Ultrafast probes of electron–hole transitions between two atomic layers

**DOI:** 10.1038/s41467-018-04751-2

**Published:** 2018-06-07

**Authors:** Xiewen Wen, Hailong Chen, Tianmin Wu, Zhihao Yu, Qirong Yang, Jingwen Deng, Zhengtang Liu, Xin Guo, Jianxin Guan, Xiang Zhang, Yongji Gong, Jiangtan Yuan, Zhuhua Zhang, Chongyue Yi, Xuefeng Guo, Pulickel M. Ajayan, Wei Zhuang, Zhirong Liu, Jun Lou, Junrong Zheng

**Affiliations:** 1 College of Chemistry and Molecular Engineering, Beijing National Laboratory for Molecular Sciences, Peking University, Beijing, 100871 China; 2 Department of Materials Science and NanoEngineering, Rice University, 6100 Main Street, Houston, TX 77005-1892 USA; 3 Beijing National Laboratory for Condensed Matter Physics, CAS Key Laboratory of Soft Matter Physics, Institute of Physics, Chinese Academy of Sciences, Beijing, 100190 China; 4 Department of Chemical Physics, University of Science and Technology of China, Hefei, Anhui 230026 China; 5 Department of Chemistry, Rice University, 6100 Main Street, Houston, TX 77005-1892 USA; 6 State Key Laboratory of Structural Chemistry, Fujian Institute of Research on the Structure of Matter, Chinese Academy of Sciences, Fuzhou, Fujian 350002 China

Correction to: *Nature Communications* 10.1038/s41467-018-04291-9, published online: 10 May 2018

The original version of this Article omitted an affiliation of Xiewen Wen: ‘College of Chemistry and Molecular Engineering, Beijing National Laboratory for Molecular Sciences, Peking University, Beijing 100871, China’. This has been corrected in both the PDF and HTML versions of the Article.

