# Supplementary material for: Ultrafast probes of electron–hole transitions between two atomic layers
Source: Nat Commun. 2018 May 10;9:1859. doi: 10.1038/s41467-018-04291-9 (PMC5945657; doi:10.1038/s41467-018-04291-9)
Supplement: Supplementary file 1 — Supplementary Information [file 41467_2018_4291_MOESM1_ESM.pdf]

Reviewers' comments:

Reviewer #1 (Remarks to the Author):

This study reports on ultrafast transient absorption measurements in Gr/MoSe2 heterostructures. The authors aim at studying the formation dynamics of interfacial quasiparticles in this system. Specifically, they compare the ultrafast behaviour in the mid-IR range of graphene, MoSe2 and the heterostructure in two different regimes. Firstly, they excite the sample by using pump pulse with energy well below the TMD energy bandgap. They observe a dip in in the transient transmission at the energy of the interfacial exciton, whose formation time is about 0.49 ps and the lifetime is 65 ps. Under this condition, graphene behaves as a photon absorber, while the TMD as a charge acceptor. Then, the system is excited by blue pulses, whose energy allows generating electron-hole pairs in the semiconductor. Even in this case, they observe the same transient feature, but the slower free carriers dynamics suggest the creation of more excitons. The study is supported by luminescence and Raman characterization measurements and by theoretical models able to describe and to support the measurements and to estimate relevant physical quantities, as excitonic internal quantum transition energy.

This paper is sound and will be of interest for scientists interested in heterostructures ultrafast dynamics and, more broadly in non-equilibrium dynamics of charge carriers and excitons in condensed matter systems. I recommend publication with the following minor comments

Comments:
• For completeness, the author should show the transient optical density maps in the addition to Fig1A-B in SI. Moreover, they should comment on the differences in the rising edge of the IR transient data, shown in Fig.1E comparing the three species.
• In pg. 12 there is a typo "Diract": it should be Dirac
• In pg. 17, referring to Gr and MoSe2 absorption at 3.1 eV the authors should either add references or show experimental data.
• In the manuscript the authors should replace "Kinetic analyses" with "Kinetic analysis".
• In pg 2 of SI, the authors assume a fixed value for the broadening of the interband transitions and the relaxation time. Although they test the effects of these two parameters, they should comment in the light of previous literature.
• In pg 9 of SI, "the result turn out" should be "the results turn out".

Reviewer #2 (Remarks to the Author):

Accept with editorial revision
Dear Authors
The work presented in the manuscript entitled "Ultrafast probes of electron-hole transitions between two atomic layers" (NCOMMS-18-02418), is very interesting and well documented. The work highlights the existence of ultrafast tightly bound interlayer excitons in the 2D hetrostructure of graphene/MoSe2 in the mid-IR region at room temperature is quiet interesting and will be of general interest to 2D-research community. Therefore, I recommend it for publication in this journal with minor correction.
Comment1: In Figure 4B (page 16), the graphene/MoSe2 structure shows the interlayer excitonic peak at 2156 cm-1, after 3.1 eV photon excitation, whereas Figure 3C shows the excitonic peak centred at 2185 cm-1 after 1.03 eV photon excitation. The shift in the peak position with high energy photon excitation needs more discussion.
Comment 2: The authors claim instantaneous thermalization of the photo-excited free carriers in graphene creates large number of holes in MoSe2, resulting interlayer excitons, through quasi-equilibrium. I suggest the authors to include the total no of excitons involved in this process for different photo excitation (e.g. for 1.03 eV and 3.1 eV).

Replies to comments:

Reviewer #1 (Remarks to the Author):

This study reports on ultrafast transient absorption measurements in Gr/MoSe2 heterostructures. The authors aim at studying the formation dynamics of interfacial quasiparticles in this system. Specifically, they compare the ultrafast behaviour in the mid-IR range of graphene, MoSe2 and the heterostructure in two different regimes. Firstly, they excite the sample by using pump pulse with energy well below the TMD energy bandgap. They observe a dip in in the transient transmission at the energy of the interfacial exciton, whose formation time is about 0.49 ps and the lifetime is 65 ps. Under this condition, graphene behaves as a photon absorber, while the TMD as a charge acceptor. Then, the system is excited by blue pulses, whose energy allows generating electron-hole pairs in the semiconductor. Even in this case, they observe the same transient feature, but the slower free carriers dynamics suggest the creation of more excitons. The study is supported by luminescence and Raman characterization measurements and by theoretical models able to describe and to support the measurements and to estimate relevant physical quantities, as excitonic internal quantum transition energy.

This paper is sound and will be of interest for scientists interested in heterostructures ultrafast dynamics and, more broadly in non-equilibrium dynamics of charge carriers and excitons in condensed matter systems. I recommend publication with the following minor comments

Comments:

"• For completeness, the author should show the transient optical density maps in the addition to Fig1A-B in SI. Moreover, they should comment on the differences in the rising edge of the IR transient data, shown in Fig.1E comparing the three species."

Reply: We assume that these should be fig.3. As suggested, the transient optical data are added into fig.S1 of SI. A sentence "Transient spectra and kinetic data are provided in SI (fig.S1)." is also added into p.8 of the main text.

"The signal of monolayer $MoSe_2$ excited with 3.1 eV photons rises apparently slower than those of samples excited with 1.03 eV photons. The difference mainly originates from different signal mechanisms. The signal with the 3.1 eV excitation directly comes from the absorptions of both fast generated free carriers and excitons generated in a subsequent slower process, but the signal with 1.03 eV excitation is from the very fast electronic thermal redistribution in graphene as discussed in the following." is added in p.9 of the main text to explain the difference in signal rising time.

“• In pg. 12 there is a typo “Diract”: it should be Dirac”

Reply: changed as pointed out.

“• In pg. 17, referring to Gr and MoSe2 absorption at 3.1 eV the authors should either add references or show experimental data.”

Reply: References 40-42 have been added to address this issue.

“• In the manuscript the authors should replace “Kinetic analyses” with “Kinetic analysis”.”

Reply: Because we have more than one analysis, “analyses” is more appropriate.

“• In pg 2 of SI, the authors assume a fixed value for the broadening of the interband transitions and the relaxation time. Although they test the effects of these two parameters, they should comment in the light of previous literature.”

Reply: This is a good point. A paragraph has been added in p.2 of SI to address this issue: “In literature[5,6], Γ lies between 0.01 and 0.06 eV while τ_e is in the range of 5 ~ 40 fs. Here, considering that electronic motions typically occur within a few fs rather tens of fs, we use the fast values Γ = 0.062 eV (500 cm^{-1}) and τ_e = 10 fs throughout the study. Nonetheless, effects of these two parameters are also tested and listed in fig.S6. It turns out that the results are not very sensitive to the parameter selections.”

“• In pg 9 of SI, “the result turn out” should be “the results turn out”.”

Reply: Changed as suggested.

Reviewer #2 (Remarks to the Author):

Accept with editorial revision
Dear Authors
The work presented in the manuscript entitled “Ultrafast probes of electron-hole transitions between two atomic layers” (NCOMMS-18-02418), is very interesting and well documented. The work highlights the existence of ultrafast tightly bound interlayer excitons in the 2D hetrostructure of graphene/MoSe2 in the mid-IR region at room temperature is quiet interesting and will be of general interest to 2D-research community. Therefore, I recommend it for publication in this journal with minor correction.

“Comment1: In Figure 4B (page 16), the graphene/MoSe2 structure shows the interlayer excitonic peak at 2156 cm-1, after 3.1 eV photon excitation, whereas Figure 3C shows the excitonic peak centred at 2185 cm-1 after 1.03 eV photon excitation. The shift in the peak position with high energy photon excitation needs more discussion.”

Reply: “The central frequency slightly redshifts from 2185 cm^{-1} with 1.03 eV excitation. This frequency difference is probably because of experimental uncertainty rather than excitonic difference. Our frequency resolution is about 9 cm^{-1}, and the broadband super continuum probe pulse has a spatial dependence of frequency distribution which can lead to the signal intensity at a certain frequency dependent on the focus condition. These two factors together can cause the peak frequency to shift for 10% of its width. Therefore, within experimental uncertainty, we would conclude that the peak frequency and width are the same as those excited with 1.03 eV photons.” is added in p.17 of the main text to discuss this issue.

Comment 2: The authors claim instantaneous thermalization of the photo-excited free carriers in graphene creates large number of holes in MoSe2, resulting interlayer excitons, through quasi-equilibrium. I suggest the authors to include the total no of excitons involved in this process for different photo excitation (e.g. for 1.03 eV and 3.1 eV).

Reply: As suggested, “The estimated exciton density is about 0.45×10^{12} cm^{-2} and 0.58×10^{12} cm^{-2} under the excitation photon energy 1.03 eV and 3.1 eV, respectively.” is added in p.13 of SI to address this issue.

REVIEWERS' COMMENTS:

Reviewer #3 (Remarks to the Author):

The authors have clarified all the raised questions. I recommend for publication.
